# Identifying Key Genes for Nasopharyngeal Carcinoma by Prioritized Consensus Differentially Expressed Genes Caused by Aberrant Methylation

**DOI:** 10.7150/jca.49392

**Published:** 2021-01-01

**Authors:** Yunqin Chen, Chun Zhou, Huabin Li, Hong Li, Yixue Li

**Affiliations:** 1 School of Life Sciences and Biotechnology, Shanghai Jiao Tong University, 800 Dong Chuan Road, Shanghai 200240, China.; 2 Center for Allergic and Inflammatory Diseases & Department of Otolaryngology, Head and Neck Surgery, Affiliated Eye, Ear, Nose and Throat Hospital, Fudan University, Shanghai 200031, China.; 3 CAS Key Laboratory of Computational Biology, CAS-MPG Partner Institute for Computational Biology, Shanghai Institute of Nutrition and Health, Shanghai Institutes for Biological Sciences, University of Chinese Academy of Sciences, Chinese Academy of Sciences, Shanghai 200031, China.

**Keywords:** integrative analysis, differently expressed genes, EBV infection, aberrant methylation, disease biomarkers

## Abstract

**Background:** Nasopharyngeal carcinoma (NPC) is an Epstein-Barr virus (EBV)-associated epithelial malignancy. Large-scale genetics or epigenetics studies of NPC have been relatively scarce and sporadic, and there are no effective targeted drugs for NPC. Integrative analysis of multiple different omics profiles has been proved to be an effective approach to shed new light on cancer.

**Methods:** We developed a pipeline to aggregate consensus differentially expressed genes (DEGs) from multiple expression datasets from different platforms. Integrated bioinformatics analysis of DNA methylation and gene expression was used to prioritize key genes in NPC. We explored the biological and clinical importance of key genes, combining differential co-expression analysis, network analysis of protein-protein and microRNA (miRNA)-target interactions, and pan-cancer survival analysis.

**Results:** We obtained 668 upregulated and 594 downregulated consensus DEGs, which enriched in the PI3K-AKT, NF-κB and immune-related pathways. In NPC, 98% of 3364 differentially methylated sites were hypermethylated. Actively expressed EBV gene *EBNA1* was positively correlated with over-expressed genes coding DNA methyltransferase and Polycomb group proteins, suggesting that EBV infection may have an important role in the hypermethylation of NPC. Through integrated analysis of DNA methylation and mRNA and miRNA expression profiles, we prioritized 56 hypermethylated downregulated genes, including 7 tumor suppressor genes, and constructed a miRNA-target regulation network consisting of 12 hypermethylated miRNAs and 25 upregulated oncogenes. The promoter hypermethylation of *PRKCB* causing its downregulation was validated by experimental results and higher *PRKCB* expression was associated with longer overall survival in head-neck squamous cell carcinoma, suggesting the potential of *PRKCB* as a promising disease biomarker for NPC.

**Conclusions:** Our integrative analysis provides reliable key genes for candidate biomarkers for diagnosis and prognosis in NPC. Based on the combined evidence of promoter hypermethylation, expression up-regulation, and association with overall survival, genes such as *SCUBE2*, *PRKCB*, *IKZF1*, *MAP4K1*, and *GATA6* could be promising novel diagnostic biomarkers, and miRNAs including *MIR150*, *MIR152*, and *MIR34* could be candidate prognosis biomarkers.

## Introduction

Nasopharyngeal carcinoma (NPC) is a malignant neoplasm that arises from the epithelium of the nasopharynx and shows remarkably skewed geographic and racial distributions. NPC is rare worldwide but common in South China, with a strikingly higher incidence 1.

NPC pathogenesis has been reported to be strongly associated with multiple factors, including host genetics, viral infection, and environmental effects, which can result in genetic and epigenetic alternations 2. A recent study showed that different subtypes of NPC could be distinguished by differences in immune cell genes, and suggested that both tumor genetics and Epstein-Barr virus (EBV) infection could influence the tumor microenvironment 3. Aberrant epigenetic alterations such as DNA methylation can disrupt or over-activate critical signaling pathways. Compared with other cancer types, such as liver, head and neck, colon, lung, thyroid, kidney, breast, pancreatic, and prostate cancers, NPC has a higher hypermethylation frequency 4, 5. Genes downregulated by promoter hypermethylation could represent biomarkers for disease progression and prognosis in NPC. However, transcriptome or epigenetics studies of NPC have been relatively scarce and sporadic. Compared with the use of a single dataset and single omics, integrative analysis of expression profiles and methylation profiles has proved to be an effective way to gain new insights and may shed more light on the molecular mechanism of carcinogenesis in NPC.

In this study, we developed a pipeline to aggregate consensus differentially expressed genes (DEGs) from multiple datasets from different platforms, including both microarray and RNA sequencing (RNA-seq) data, and combined methylation profiles and microRNA (miRNA) expression profiles to identify aberrantly methylated DEGs or DEGs regulated by differentially methylated (DM) miRNAs. We prioritized DEGs using voting and robust rank aggregation (RRA) 6 methods and identified reliable DEGs based on votes and RRA-adjusted p-values. As the activation of oncogenes and loss of tumor suppressor genes (TSGs) have important roles in cancer evolution, oncogenes and TSGs were investigated to select likely biomarkers and therapeutic targets for NPC. Furthermore, owing to the lack of survival information for the sample and the availability of The Cancer Genome Atlas (TCGA) data, pan-cancer analysis of the associations between gene expression and survival was also performed to determine the biological importance of these genes.

## Materials and Methods

### Data sources for expression and methylation profiles of NPC

We searched for “nasopharynx cancer” in EBI ArrayExpress (https://www.ebi.ac.uk/arrayexpress/) and manually selected expression and methylation profiles of NPC. To identify biomarkers discriminating cancer from normal tissue, we studied human tissue samples using a cancer vs. cancer-free control design. Finally, six mRNA expression datasets and two methylation datasets were screened out for analysis (Table 1). To investigate differences in expression between aberrantly hypermethylated miRNAs in cancer and those in normal tissue, three miRNA expression datasets for more than 50 patients were used. Two additional datasets, GSE102349 and GSE103611, were used for interpretation or validation of our analysis results. GSE102349 contained both EBV and human expression profiles by RNA-seq from113 undifferentiated nasopharyngeal carcinoma tumors (no normal controls) and can be used to identify co-expressed EBV-host gene pairs in NPC. GSE103611 provided expression profiles for 24 NPC tumor tissues with distant metastasis after radical treatment and 24 without distant metastasis, and was used to annotate genes associated with distant metastasis.

### Prioritizing DEGs in NPC from multiple studies and identifying reliable consensus DEGs

The R software 7 version 3.5.1 was used for data analysis. Different strategies were applied to integrate the six mRNA expression datasets from microarrays (Affymetrix and Agilent) and RNA-seq, crossing four platforms (Table 1).

Of the four Affymetrix datasets, three using the sample platform GPL570 were combined into one integrated dataset S1. S2 planned to be only GSE13597, which had only 3 normal and 25 cancer samples, and used the platform GPL96. However, to achieve sufficient power to get more reliable DEGs, we merged S1 and GSE13597 into S2 (Supplementary Figure 1A). During data integration, the merged dataset was normalized and batch effects were removed using the ComBat function from 'sva' package 8 (Supplementary Figure 1B). S1 contained 20188 genes and 76 samples (18 controls and 58 NPC), and S2 contained 12403 genes and 104 samples (21 controls and 83 NPC).

S1, S2, S3 (Agilent), and S4 (RNA-seq) were used for DEGs identification. DEGs from microarrays were identified using the “limma” package 9, whereas those from RNA-seq were called using the “DESeq2” package 10. We used STAR 11 to map RNA-seq reads to reference genome hg19. *p*-values were adjusted using the Benjamini-Hochberg (BH) method 12. The criteria for DEGs were: 1) adjusted p<0.05; 2) absolute fold change > 2 between cancer and normal tissue.

Meta-analysis was used to aggregate the DEGs from each dataset. We aggregated upregulated and downregulated DEGs, respectively, using two methods: voting and RRA 6. The vote number is the number of datasets in which genes are significantly differentially expressed. As detectable genes were not the same across the four platforms, RRA was used to pick out DEGs that were markedly changed according to only one study but not detected in other studies as data were not available. RRA scores are negatively correlated with vote number (Spearman's rho = -0.7 for upregulated genes and -0.55 for downregulated genes, *p*<0.01). A DEG with vote number ≥2 or RRA BH-adjusted *p*<0.01 was considered reliable and used for further analysis.

### Differential co-expression analysis of DEGs

DEG expression profiles from S1 were used for the differential co-expression analysis, because the gene number of S1 was largest among the 3 datasets with #normal sample >10. R package DCGL 13 was used to find differentially co-expressed gene pairs among the DEGs. Differential co-expression profile and differential co-expression enrichment methods were used to identify differentially co-expressed genes (DCGs) and differentially co-expressed links (DCLs). DCLs whose absolute correlation coefficients were not less than 0.5 in at least one situation (normal or cancer) were selected. To check types of link change, we classified DCLs into three types: “loss-of-association”, “gain-of-association”, and “reverse-of-association”. If the absolute correlation coefficient of a DCL in cancer was significantly stronger than that in the normal condition, it would be grouped into the “gain-of-association” type, if the reverse was true, it was considered to belong to the “loss-of-association” type. The “reverse-of-association” type was the case where the direction of the relationship of DCL switched between cancer and the normal condition.

### DEGs probably regulated by aberrant methylated promoters or miRNAs

**Figure 1:**
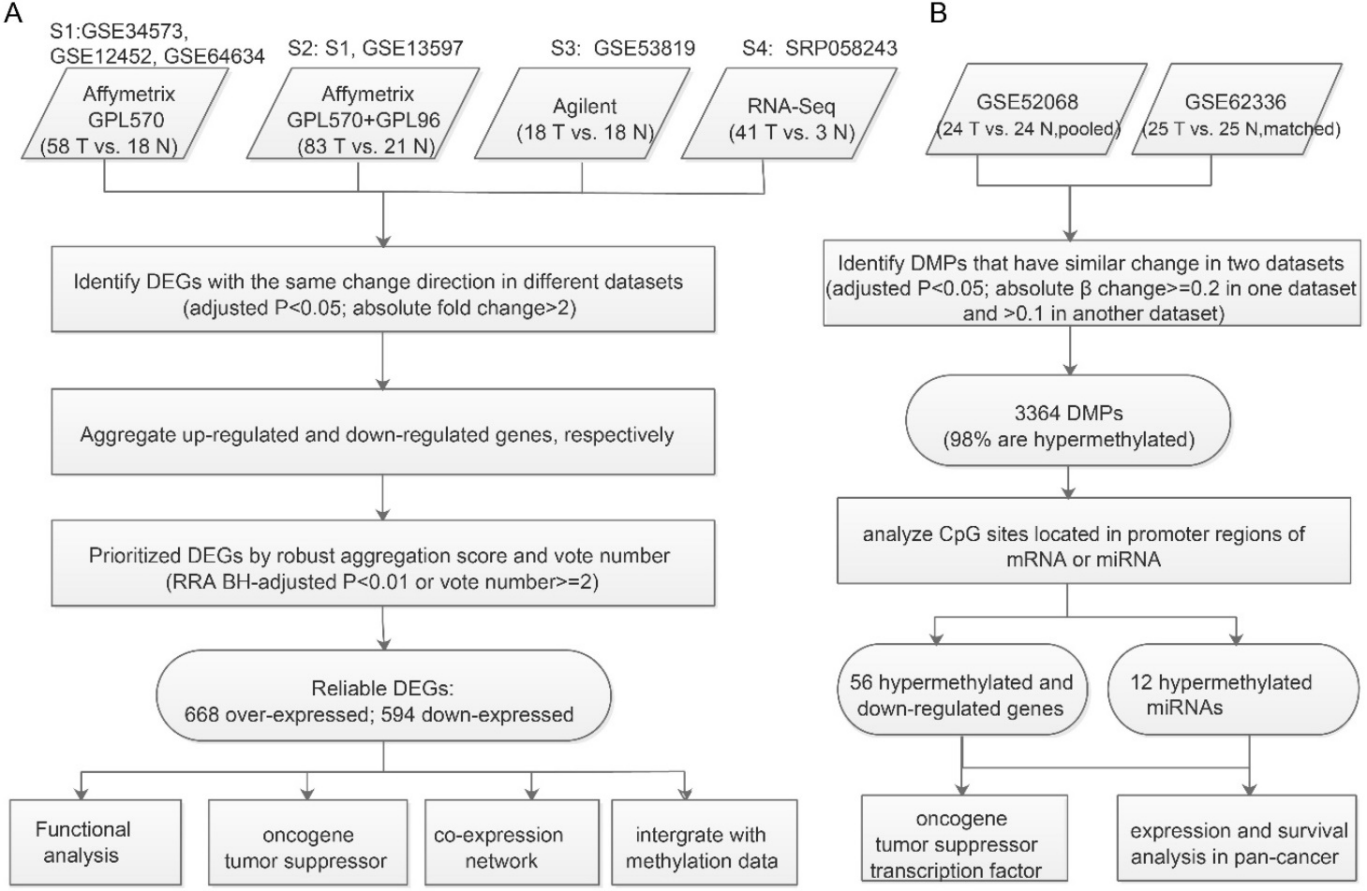
** Workflow for identifying prioritized DEGs caused by aberrant methylation. A.** Integrated analysis of multiple expression profiles to prioritize DEGs. **B.** Pipeline for identifying aberrantly methylated DEGs and DM miRNAs-DEGs.

For we didn't have matched samples with both expression and methylation profiles, we identify DEGs likely caused by aberrant Methylation through applying the strategy of 'overlapping'. The “limma” package 9 was used to identify DM probes (DMPs). Both the methylation datasets used Illumina HumanMethylation450 BeadChip, but GSE62336 had matched normal controls, for which paired comparison design was appropriate, whereas GSE52068 used pooled normal controls and so pooled comparison design was preferable. DMPs were selected using the following cutoff: 1) adjusted *p*<0.05; 2) absolute β change ≥0.2 in one dataset and > 0.1 in another. DMPs were annotated using the package IlluminaHumanMethylation450kanno.ilmn12.hg19 14. The promoter region was defined as 1500bp before the TSS of each gene plus 5' UTR. If the promoter region of a gene or miRNA had DMPs, we considered it to be a DMG or DM miRNA. Recurrent DMGs or DM miRNAs (identified in both datasets and with the same direction of change) were used to overlap with consensus DEGs to identify DEGs likely caused by aberrant methylation (**Figure 1B**).

Hypomethylated upregulated or hypermethylated downregulated DEGs and over-expressed DEGs targeted by hypermethylated miRNAs or under-expressed DEGs targeted by hypomethylated miRNAs were considered to be aberrant methylation-regulated DEGs.

### Gene function enrichment analysis, transcription factors, and cancer gene annotation

We used the R package clusterProfiler 15 to perform gene ontology and pathway enrichment analysis. Adjusted *p*-values less than 0.01 were obtained by the BH method were regarded as statistically significant. The human transcription factor list was downloaded from the supplementary file of Lambert et al. 16.

The oncogene list was obtained from the ONGene database 17 (http://ongene.bioinfo-minzhao.org/), and the tumor suppressor genes (TSGs) list was from the TSGene database 18 (https://bioinfo.uth.edu/TSGene/index.html). The activation of oncogenes and inactivation of TSGs may be associated with the cancer development.

### Protein-Protein Interaction (PPI) and miRNA-target interactions (MTIs)

PICKLE (Protein InteraCtion KnowLedgebasE) is a meta-database for the human direct protein-protein interactome, integrating publicly available PPI databases via genetic information ontology 19. A total of 179738 standard and cross-checked PPIs were downloaded from PICKLE. miRNA-target interactions (MTIs) from the experimentally validated MTI database miRTarBase are validated experimentally by reporter assay, western blot, microarray and next-generation sequencing experiments 20. A total of 502652 human MTIs (containing 2599 miRNAs and 15050 target genes) were obtained from miRTarBase.

### Survival analysis of hypermethylated downregulated genes and miRNAs using TCGA data

TCGA PanCanAtlas gene and miRNA expression and patient survival information were obtained from the NCI Genomic Data Commons Data Portal (https://gdc.cancer.gov/about-data/publications/pancanatlas). For tumor versus normal tissue comparisons, we only considered cancer types with more than 10 normal samples. The Cox regression model and log-rank test were used to determine prognostic power.

### DAC treatment, RNA isolation and qRT-PCR

Human nasal epithelial cells (hNEPC) and NPC cell line C666-1 were planted to 6-well-plate, after growing for 24 hours, the cells were treated with vehicle (DMSO) or 10 mM DAC (5-aza-2'-deoxycytidine, MCE NSC 127716). The medium containing DMSO/DAC were changed every 24 hours for 72 hours. After treatment, total RNAs were extracted using TRIzol (Invitrogen Cat No.:10296010) according to the manufacturer's instruction. cDNA was reversely transcribed from 500 ng total RNA by PrimeScript RT-PCR kit (Takara Cat No.: RR014). Real-time PCR was carried out on an ABI 7900HT Fast Real-Time PCR System. Data shown were the relative abundance of the indicated mRNA normalized to that of Gapdh by the change-in-cycling-threshold (∆∆CT) method. The primers for real-time PCR were listed in the Supplementary Table 1.

## Results

### Identification and Characteristics of consensus DEGs

We collected all available qualified expression and methylation profiles for NPC including six mRNA expression datasets (Table 1). To integrate mRNA expression profiles from different studies and platforms we first merged datasets from the same platform by unique gene ID and removed batch effects using the ComBat method 21. After merging, four datasets from four platforms were used to determine DEGs between NPC and normal tissues. The analysis pipeline for expression and methylation data is illustrated in Figure 1.

**Figure 2:**
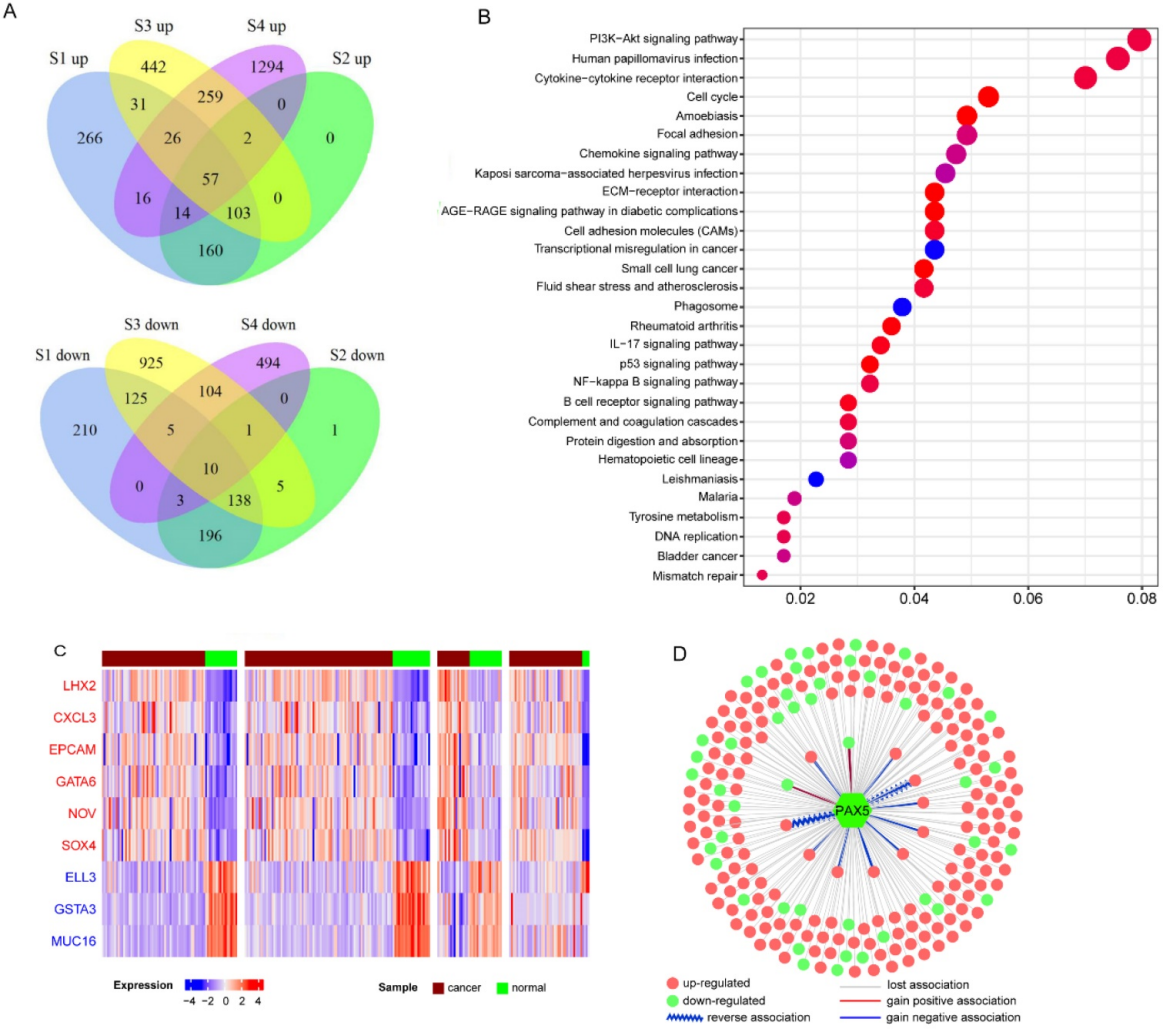
** Characteristics of consensus DEGs. A.** Venn diagram of DEGs identified from each dataset; “up” means upregulated DEGs, “down” means downregulated DEGs. **B.** Functional enrichment of consensus DEGs. **C.** Heatmap of top-ranked upregulated and downregulated DEGs. **D.** DCLs of *PAX5* in cancer.

In total, 2670 upregulated genes and 2217 downregulated genes were identified at least one dataset (Figure 2A). Then we used meta-analysis to integrate DEGs from different platforms, using a combination of vote number and RRA to obtain consensus DEGs (see Materials and Methods). If a DEG had vote number ≥2 or RRA-adjusted *p*<0.01, it was considered to be reliable and used for further analysis. Finally, 1261 DEGs were obtained, including 668 upregulated and 594 downregulated genes (gene *S100A2* was downregulated in S3 and S4 but over-expressed in S1 and S2). Functional enrichment analysis of all DEGs revealed that they were enriched in cancer-related pathways including the PI3K-AKT signaling pathway, NF-κB signaling pathway, p53 signaling pathway, focal adhesion and immune-related pathways, chemokine signaling pathway, IL-17 signaling pathway, and B cell receptor signaling pathway (Figure 2B). Virus-associated pathways (Kaposi sarcoma-associated herpesvirus infection and human papillomavirus infection) were also over-represented.

To further understand the functions of DEGs, we annotated DEGs with oncogenes, TSGs, and transcription factors, and ranked them by vote number and RRA score (Supplementary Table 2; see Materials and Methods). Among the 1261 DEGs, there were 60 over-expressed oncogenes and 52 downregulated TSGs. Oncogenes *CXCL3*, *EPCAM*, *GATA6*, *NOV*, and *SOX4* were upregulated in all four datasets (Figure 2C), indicating their important roles in NPC carcinogenesis. *LHX2* was the top upregulated gene based on RRA score. *LHX2* is a transcription factor and a mesenchymal marker of epithelial-mesenchymal transition (EMT), which has been reported to promote tumor growth and metastasis in pancreatic ductal adenocarcinoma and breast cancer 22, 23. The top three downregulated genes were *ELL3*, *GSTA3*, and *MUC16* (Figure 2C). Both *GSTA3* and *MUC16* have protective roles against EMT 24, 25. Additionally, through integrating the dataset with primary and metastatic NPC (GSE103611), we identified 11 up-regulated DEGs (including oncogenes like SOX4, FNDC3B and JUP, and EMT regulatory factors like *SNAI2*) and 2 down-regulated DEGs (immune-related genes *CR2* and *LAT2*) associated with distinct metastasis (Supplementary Table 2).

Next, we constructed co-expression networks of DEGs using the expression profiles in NPC and normal samples, respectively (see Materials and Methods). Differential co-expression analysis was performed to investigate changes in gene-gene links; 388 DCGs and 33509 DCLs were identified. Our results showed that almost 96.3% DCLs lost associations, 3.2 % gained new associations, and only 0.4% showed reversed associations (Supplementary Figure 2A). On the other hand, 288 (74.2%) DCGs were downregulated in NPC, significantly more than in the background (47% DEGs were downregulated, chi-square test p<0.01). These results indicate that the co-expression network of NPC has a trend of loss-of-association of downregulated genes, which may result in the dysregulation of normal gene links, especially silencing of TSG regulation. For example, TSG *PAX5* is a B cell transcription factor, which is downregulated in NPC. *PAX5* restoration can cause rapid repression of Myc and DNA replication factor 26. In our study, 95% of 227 DCLs of *PAX5* were of the loss-of-association type, and 83% differentially co-expressed partners (DCPs) were upregulated in NPC, probably owing to loss-of-regulation of *PAX5* (Figure 2D and Supplementary Figure 2B). Functional enrichment analysis suggested that PAX5 DCPs were enriched in the cell division biological function and the DNA replication signaling pathway.

### Global hypermethylation and association with EBV gene expression

**Figure 3:**
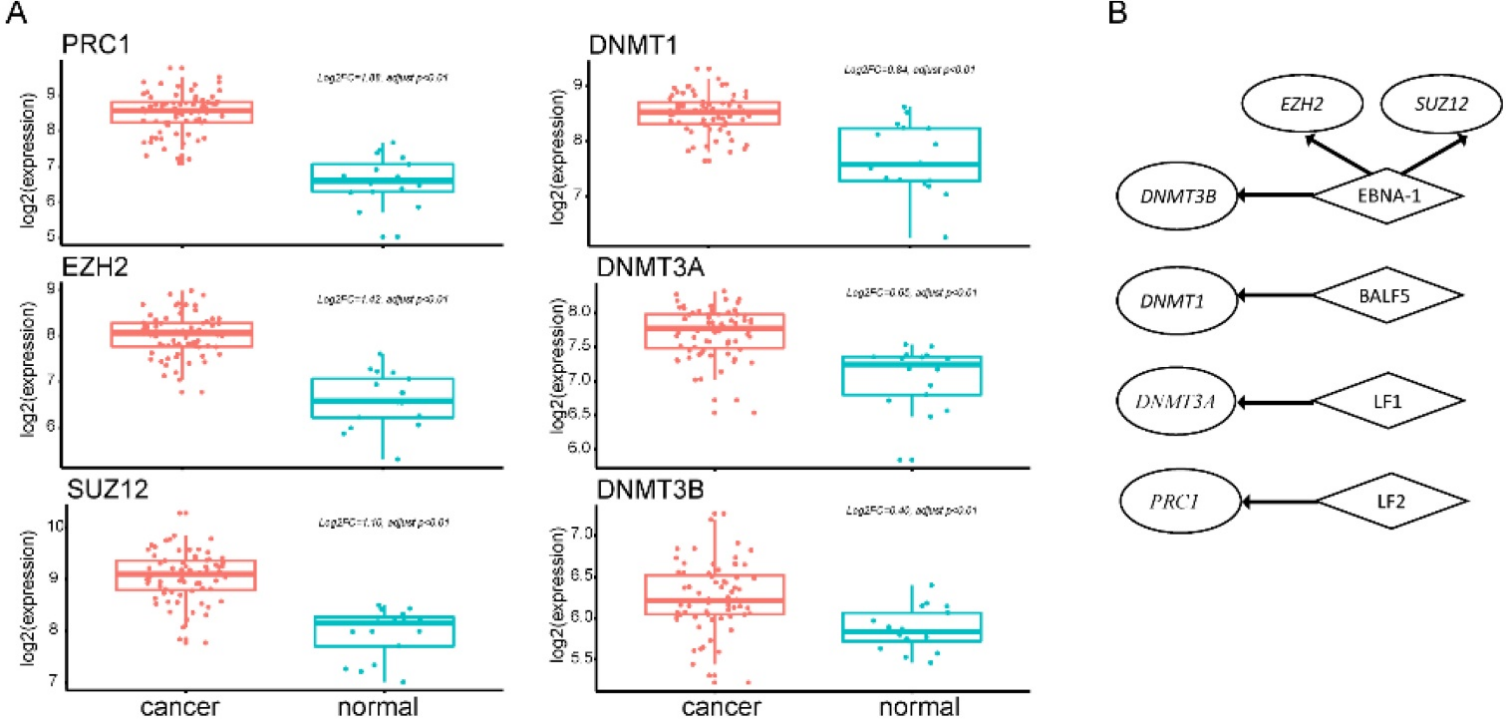
** Ectopic expression of hypermethylation-associated genes and their association with EBV infection. A.** Upregulated genes of DNA methyltransferase and PcG proteins in NPC. **B.** Significantly positively correlated gene pairs between actively expressed EBV genes and epigenetic regulator genes. Human epigenetic regulator genes were italicized and shown in ellipse while EBV genes were not italicized and shown in diamond.

EBV is an important risk factor for NPC. EBV infection can cause epigenetic changes in the host genome and promote tumorigenesis. Previous studies have shown that EBV-associated tumors are globally hypermethylated. Here, we identified 3364 DM sites, and 3284 (98%) were hypermethylated (Figure 1B). Therefore, we tried to explore the molecular mechanism between EBV and hypermethylation. DNA methyltransferase and the Polycomb group (PcG) family proteins are critical to DNA methylation levels and often cooperate in silencing gene expression. We found that genes coding DNA methyltransferase (*DNMT1*, *DNMT3A*,* DNMT3B*) and PcG proteins (*PRC1*, *EZH2*, *SUZ12*) were over-expressed in NPC (Figure 3A). Analysis of RNA-seq dataset S4, which contained both host and EBV transcriptomes, showed that seven lytic genes (*BALF3*, *BALF4*, *BALF5*, *BILF1*, *LF1*, *BARF1*, *LF2*), two latent genes (*LMP-1*, *LMP-2B*), and* EBNA-1* were actively expressed in NPC patients. The lytic EBV gene products may directly induce DNA damage and contribute to NPC development. To explore whether EBV gene expression programs were associated with host gene expression of DNA methyltransferase and PcG proteins, we performed a correlation analysis between human genes and the top 10 highly expressed EBV genes. The significantly positively correlated pairs are shown in Figure 3B (Pearson's correlation coefficient >0.3, *p*<0.05). *EBNA1* was positively associated with both kinds of genes, which was confirmed by other study using dataset GSE102349 3 (Supplementary Table 3). EBNA1 is the only nuclear EBV protein expressed in both latent and lytic modes of infection, and is required for the replication and maintenance of the episomal EBV genome. EBNA1 is significantly correlated with *EZH2*, *SUZ12*, and *DNMT3B*, indicating a potential role for *EBNA1* in the global hypermethylation of NPC.

### Downregulated genes induced by promoter hypermethylation

Hypermethylation in gene promoters is a well-known mechanism for the silencing of TSGs. We found 56 hypermethylated and downregulated genes in NPC (Supplementary Table 2). PPI data from PICKLE were used to investigate the interactions of these 56 genes and other genes. Functional enrichment analysis of all genes in the PPI network showed that they were enriched in the following immune-related pathways: “T cell receptor signaling pathway”, “Natural killer cell mediated cytotoxicity”, “Epstein-Barr virus infection”, and “TNF signaling pathway” (Supplementary Table 4).

Among the 56 hypermethylated and downregulated genes, there were seven TSGs (Table 2). These silenced TSGs are important for the carcinogenesis of NPC. Previous studies have reported that hypermethylation of *HOPX*, *IRF8*, and *SHISA3* caused downregulation of gene expression and promoted the metastasis of NPC 27-29. Although the roles of another four genes (*SCUBE2*, *PRKCB*, *IKZF1*, and *MAP4K1)* in NPC were not known, their functions were reported in other cancer types. *SCUBE2* is silenced by CpG island hypermethylation in breast cancer, and its activation could inhibit cancer cell migration and invasion through the reversal of EMT 30. Transcription factor *IKZF1* is a critical regulator of lymphoid differentiation and its encoding protein Ikaros regulates the development and function of the immune system 31. *PRKCB* and *MAP4K1* are kinases and participate in many signaling pathways.

**Figure 4:**
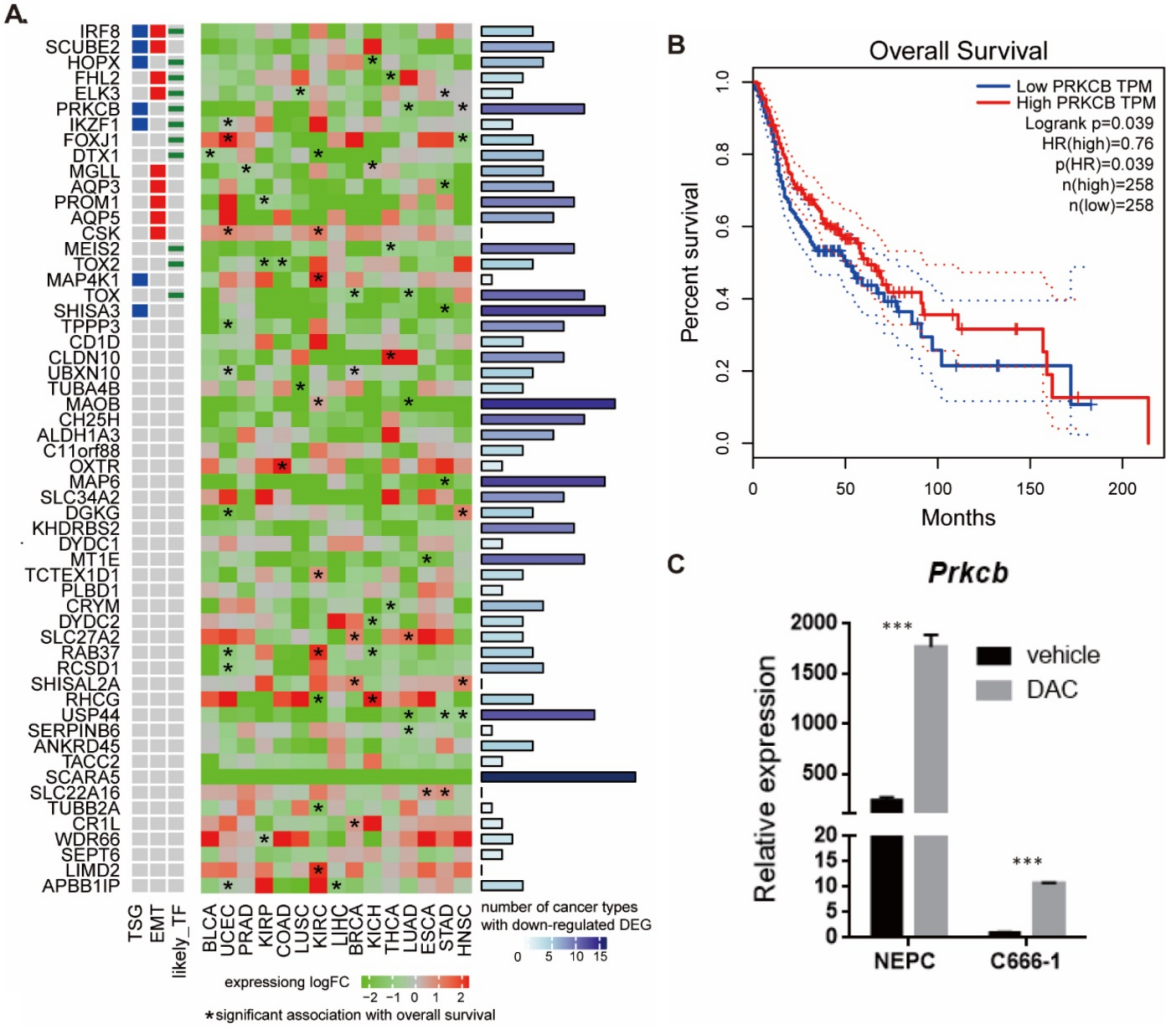
** Pan-cancer expression and survival analysis of 56 hypermethylated downregulated genes. A.** Pan-cancer expression and survival analysis of 56 hypermethylated downregulated genes. **B.** Higher *PRKCB* expression was associated with longer overall survival in HNSC. **C.** *PRKCB* down-expressed in NPC cell line and PRKCB expression was upregulated after DAC treatment.

To better understand the biological role of the 56 hypermethylated and downregulated genes in cancer, we used data from TCGA to investigate their expression changes and relationship with survival (Figure 4A). We found that most of the 56 genes were downregulated in other cancers, especially in head-neck squamous cell carcinoma (HNSC). *SCARA5*, *MAOB*, *MAP6*, *SHISA3*, *USP44*, *CH25H*, *TOX*, and *PRKCB* were downregulated in at least 10 types of cancer. Expression of some genes tended to positively correlate with overall survival (hazard ratio <1, *p*<0.05). The pan cancer analysis indicates the potential functional roles of these genes in NPC.

*PRKCB* is both tumor suppressor gene and transcriptional factor and higher *PRKCB* expression was associated with longer overall survival in HNSC (Figure 4B). To validate the relationship between expression of gene and methylation of its promoter in NPC, we examined the expression levels of *PRKCB* before and after treatment with the demethylating drug DAC using human nasal epithelial cells (hNEpC) and NPC cell line C666-1 (see Methods). qRT-PCR revealed that the mRNA level of *PRKCB* was significantly lower in NPC cell line C666-1 than hNEPC. After DAC treatment, the expression level of* PRKCB* statistically increased in C666-1 (*p*<0.001) (Figure 4C), which suggested *PRKCB* downregulation in NPC was due to its promoter hypermethylation.

### Oncogenes regulated by hypermethylated downregulated miRNAs through MTIs

We found twelve miRNAs whose promoters were hypermethylated in NPC in both methylation datasets. To check whether hypermethylation in the miRNA promoters could lead to decreased expression, we analyzed three miRNA expression datasets with more than 50 patients: GSE32960, GSE36682, and GSE70970 (Table 1). Eight of the twelve hypermethylated miRNAs showed significantly lower expression in NPC than in normal tissue in at least one of the expression datasets (Supplementary Table 5).

**Figure 5:**
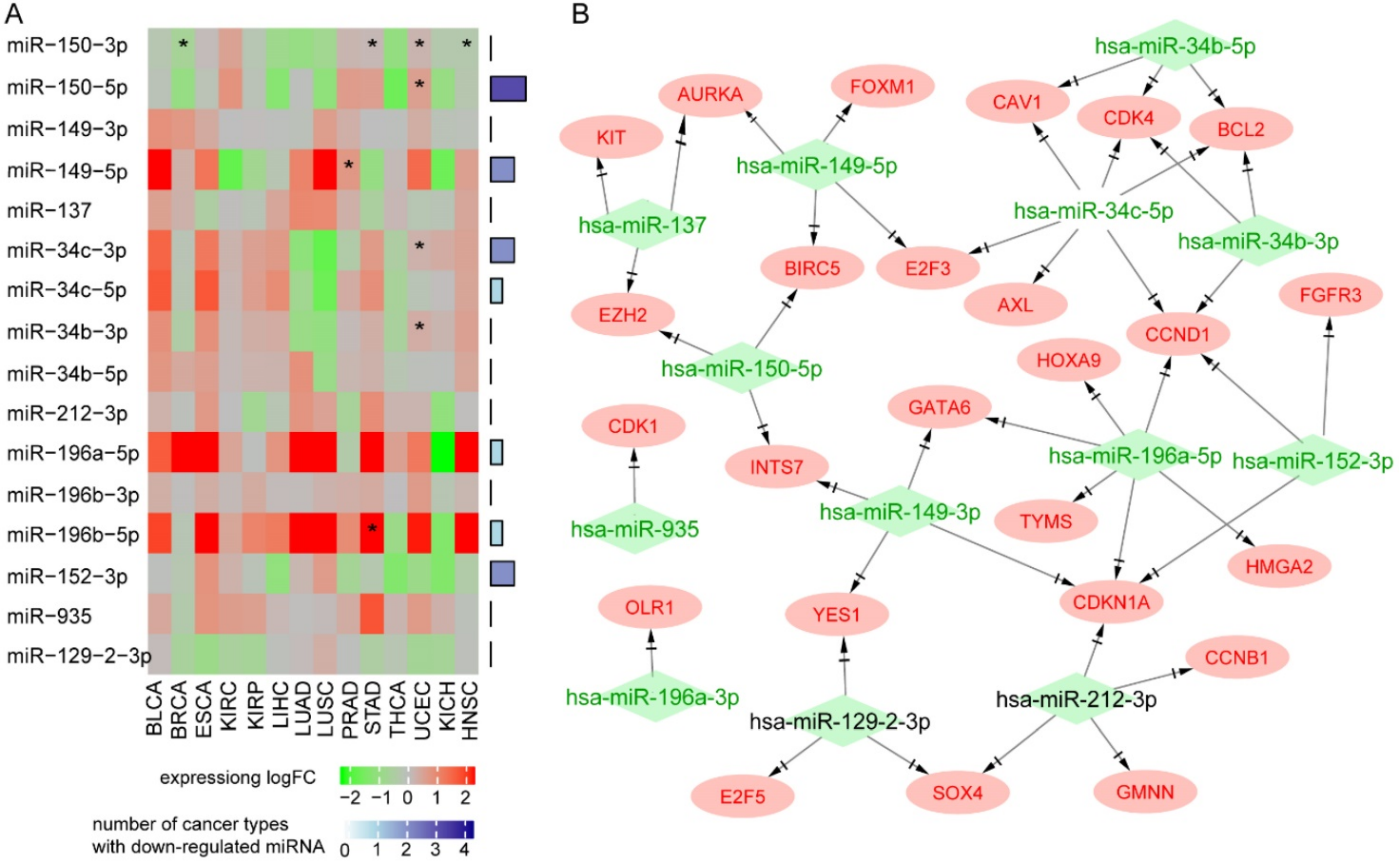
** Oncogenes regulated by hypermethylated downregulated miRNAs through MTIs. A.** Pan-cancer expression and survival analysis of 12 hypermethylated miRNAs. **B.** Regulation network of hypermethylated miRNAs and their targeted upregulated oncogenes.

Pan-cancer analysis showed that *MIR129-2*, *MIR149*, *MIR152*, *MIR150*, *MIR34B*, and *MIR34C* were downregulated in at least four types of cancer, and *MIR150* had a protective role against death in most types of cancer (Figure 5A). Seven miRNAs (*MIR129-2*,* MIR149*, *MIR152*, *MIR137*, *MIR150*, *MIR34B*, and *MIR34C*) have been reported as tumor suppressors in many types of cancer. Upregulation of the *miR-34* family resulted in apoptosis and cell-cycle arrest through targeting p53 32, 33. *MIR152* and *MIR137* inhibited cell proliferation, migration, and invasion in human cancers 34-37. Downregulation of *MIR129-2* by promoter hypermethylation induced breast cancer cell proliferation and apoptosis 38. *MIR149* plays an important part in tumorigenesis and tumor progression, and its downregulation promoted the metastatic dissemination of tumor cells by supporting aberrant Rac activation in breast cancer 39. Survival analysis of 312 NPC patients from GSE32960 showed that patients with higher *MIR150*, *MIR137*, or *MIR152* had a better survival rate (log rank test, *p*<0.05, data not shown), suggesting that they also act as tumor suppressors in NPC.

Next, we explored the effects of 12 hypermethylated miRNAs on their target genes. As miRNAs generally repress gene expression, our hypothesis was that hypermethylation of a miRNA promoter would reduce its expression and thus increase the expression of its target genes. We found 181 MTIs between the 12 miRNAs and 128 over-expressed target genes (Supplementary Table 6). Functional analysis of 128 target genes showed enrichment in the following cancer signaling pathways: “cell cycle”, “PI3K-Akt signaling pathway”, and “p53 signaling pathway”. Interestingly, *PDL1* was highly expressed in NPC and regulated by hypermethylated *MIR152* and *MIR34b*, indicating the potential for immunotherapy using agents mimicking miR-152 or miR-34 to inhibit PD-L1 and enhance T cell proliferation and effector cytokines.

Among the 128 over-expressed target genes, 25 were oncogenes, forming a miRNA-oncogene network with 43 links (Figure 5B). Several oncogenes were regulated by multiple miRNAs. Oncogenes *CCND1* and *CDKN1A* and transcription factor Homeobox C8 (*HOXC8*) were regulated by four miRNAs. Ectopic expression of *HOXC8* can modulate NPC cell growth *in vitro* and *in vivo*, and EBV gene LMP1 represses *HOXC8* 40. Epigenetic regulator *EZH2* was regulated by tumor suppressor *MIR150* and *MIR137*. Transcription factor *SOX4* was over-expressed in all expression datasets and regulated by *MIR129* and *MIR212*. The expression of *MIR212* has been reported to be decreased in NPC tissues and cells, and its overexpression could inhibit the metastasis of nasopharyngeal carcinoma by targeting *SOX4* 41; patients with higher expression levels of *SOX4* had poorer survival rates 42.

## Discussion

NPC is poorly understood at the genetic level, and there is an urgent need for more efficient approaches such as targeted therapy and immunotherapy. There have been few studies of the genomic changes in NPC. Lin et al. in 2014 found that enrichment of genetic lesions in NPC affected several important cellular processes and pathways, including chromatin modification, ERBB-PI3K signaling, and autophagy machinery 43. Li et al. in 2017 also identified NF-κB pathway-activating mutations during cancer pathogenesis 44. Different subtypes of NPC harbored different genomic alterations, and a high frequency of *IHD2* mutations was detected in undifferentiated NPC 45. However, a comprehensive and systemic study of gene expression and epigenetic changes was still lacking; this will be helpful to better understand how cancer evolves and find candidate biomarkers for NPC.

In our work, through integrating multiple types of datasets using bioinformatics analysis, we identified reliable consensus DEGs. Considering the difference in detectable genes among different platforms, we selected not only recurrent DEGs but also the top genes with markedly differential expression in only one study using the RRA method.

Furthermore, we investigated the interaction between DEGs using differential co-expression analysis, and found that the majority of DCGs were downregulated and tended to lose association in cancer; 98% of DCPs that lost positive correlations with *PAX5* were upregulated, whereas 85% of DCPs that lost negative correlations were downregulated (Supplementary Figure 1B). This suggested that owing to lost associations, downregulated TSG *PAX5* lost regulation of its target genes, and some mis-regulated oncogenes were overexpressed.

NPC is distinguished by differences in immune cell genes, and both tumor genetics and EBV infection influence the tumor microenvironment 3. Upregulated genes of DNA methyltransferase and PcG proteins were co-expressed with actively expressed EBV genes, especially *EBNA1*, suggesting EBV interacts with the host genome and affects host genome methylation, thereby causing NPC.

Combining epigenetic data, we found DEGs regulated by aberrant methylation in gene promoter regions or DM miRNAs. 7 downregulated TSGs with aberrant hypermethylation in promoters and 43 upregulated oncogenes regulated by hypermethylated miRNAs were potential biomarkers and therapeutic targets for the precise diagnosis and treatment of NPC. Our work did not analyze survival rates and prognoses owing to the unavailability of clinical data; however, pan-cancer analysis of 14 cancers in TCGA showed that hypermethylated and downregulated *MAP4K1*, *PRKCB*, *IKZF1*,* MAP4K1*, and *SHISA3* were associated with longer overall survival in HNSC and other cancers. Furthermore, our experimental results validated that *PRKCB* downregulation in NPC was caused by its promoter hypermethylation. Dysregulated transcription factor *PRKCB* could be an appropriate target for the development of anticancer drug 46.

## Conclusion

Through comprehensive integrative analysis of different types of data, we have shed more light on the molecular mechanism of carcinogenesis in NPC and provided disease biomarkers for NPC and the hypermethylation of *PRKCB* could be a novel and promising diagnosis marker.

## Supplementary Material

Supplementary figures.

Supplementary table S1-S6.

## Figures and Tables

**Table 1:** Expression and methylation datasets analyzed and integrated in the study

Technology	Platform	Raw Dataset	#Sample^a^	Design	EBV infection	DEGs dataset
Microarray	GPL570 (HG-U133_Plus_2)	GSE34573	19(4+15)	Unpaired	EBV+	S1
Microarray	GPL570 (HG-U133_Plus_2)	GSE12452	41(10+31)	Unpaired	EBV+	
Microarray	GPL570 (HG-U133_Plus_2)	GSE64634	16(4+12)	Unpaired	Unknown	
Microarray	GPL96 (HG-U133A)	GSE13597	28(3+25)	Unpaired	EBV+	S2*
Microarray	GPL6480 (Agilent-014850 )	GSE53819	36(18+18)	Unpaired	Unknown	S3
RNA-seq	Illumina Hiseq 2000	SRP058243	45(4+41)	Unpaired	EBV+	S4
Methylation array	Illumina HumanMethylation450 BeadChip	GSE52068	48(24+24)	Unpaired	Unknown	/
Methylation array	Illumina HumanMethylation450 BeadChip	GSE62336	50(25+25)	Paired	Unknown	/
miRNA array	GPL14722	GSE32960	330(18+312)	Unpaired	EBV+	/
miRNA array	GPL15311	GSE36682	68(6+62)	Unpaired	EBV+	/
miRNA array	GPL20699	GSE70970	264(17+246)	Unpaired	EBV+	/

^a^ Sample: n (normal + cancer); *for enough power to calculate DEGs, S2 included GSE13597 and S1.

**Table 2:** Seven downregulated hypermethylated tumor suppressor genes in NPC

Gene	Transcriptional factor	TF-domain	EMT-related	Comments*
*SCUBE2*	NO	-	Yes	Novel
*HOPX*	YES	Homeodomain	-	Known
*IRF8*	YES	IRF	Yes	Known
*PRKCB*	YES	Unknown	-	Novel
*IKZF1*	YES	C2H2 ZF	-	Novel
*MAP4K1*	NO	-	-	Novel
*SHISA3*	NO	-	-	Known

*Novel: not reported in previous study. Known: validated by other studies.

## Abbreviations

NPC: Nasopharyngeal Carcinoma
EBV: Epstein-Barr virus
DEG: Differentially Expressed Genes
DM: Differentially Methylated
RRA: Robust Rank Aggregation
TSG: Tumor Suppressor Gene
TCGA: The Cancer Genome Atlas
PPI: Protein-Protein Interaction
MTI: miRNA-target interaction
hNEPC: Human nasal epithelial cells
DAC: 5-aza-2'-deoxycytidine
