# Supplementary figures and images for: Identifying Key Genes for Nasopharyngeal Carcinoma by Prioritized Consensus Differentially Expressed Genes Caused by Aberrant Methylation
Source: J Cancer. 2021 Jan 1;12(3):874–84. doi: 10.7150/jca.49392 (PMC7778547; doi:10.7150/jca.49392)

A

### Transcriptomics data cohort (S2)

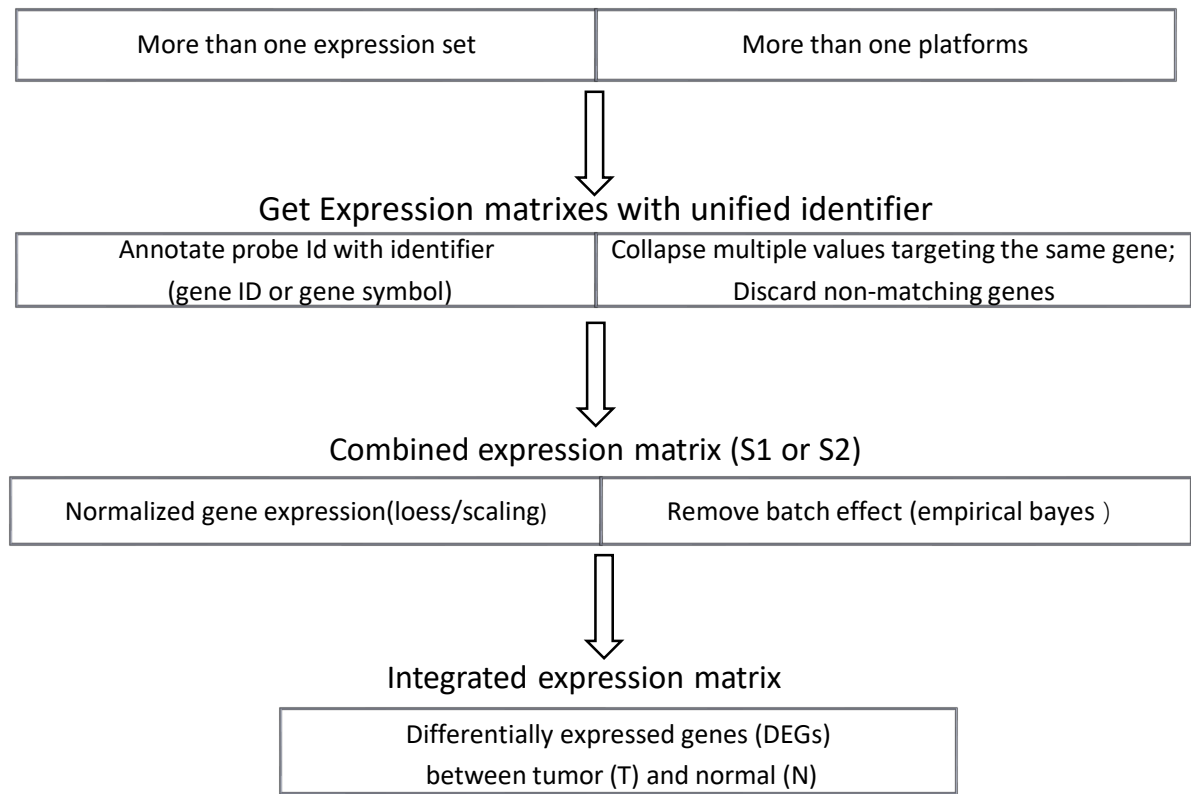

B

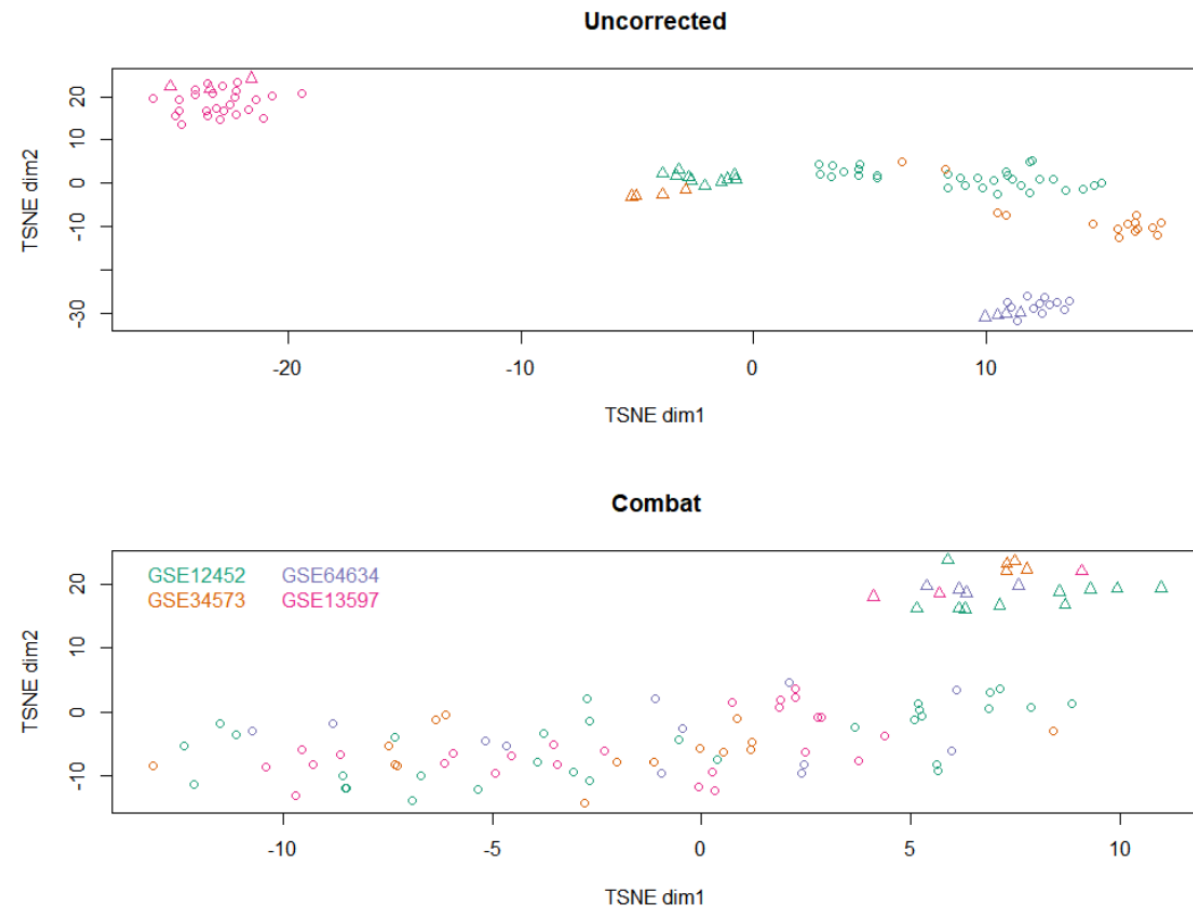

**A**

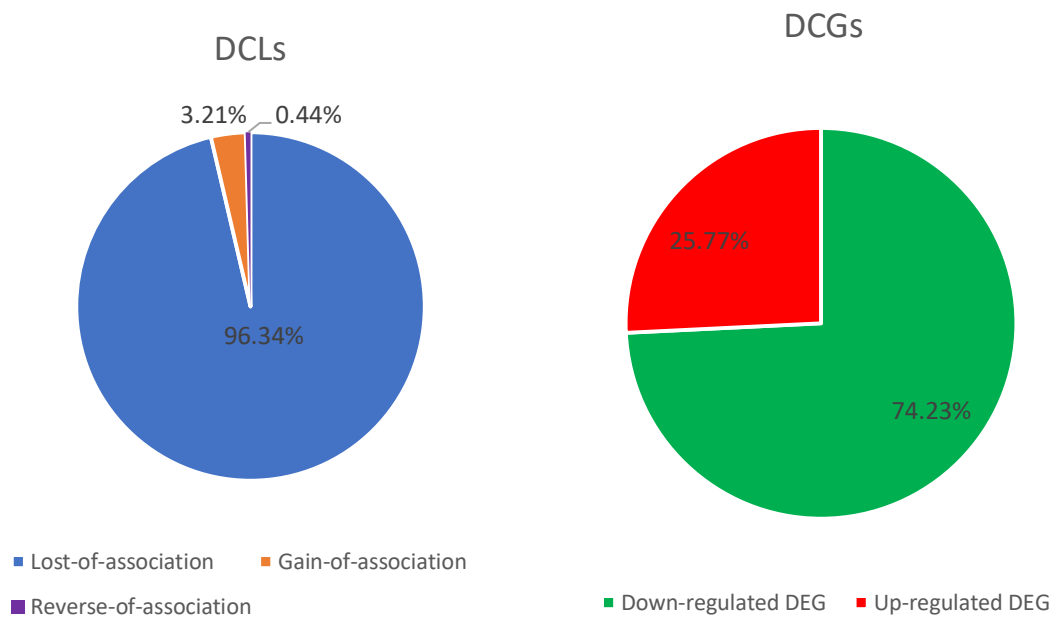

**B**

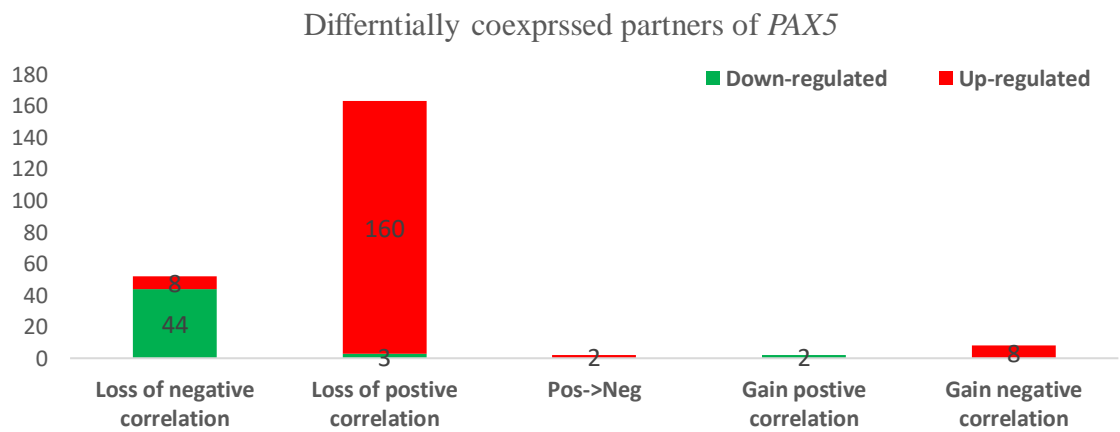

Supplement: Supplementary file 1 — Supplementary figures. [file jcav12p0874s1.pdf]
